# Carbohydrates as enantioinduction components in stereoselective catalysis

**DOI:** 10.1039/c6ob00368k

**Published:** 2016-03-31

**Authors:** Alexander S. Henderson, John F. Bower, M. Carmen Galan

**Affiliations:** a School of Chemistry , University of Bristol , Cantock's Close , Bristol. BS8 1TS , UK . Email: John.Bower@bristol.ac.uk ; Email: M.C.Galan@bristol.ac.uk

## Abstract

Carbohydrate derivatives are readily available chiral molecules, yet they are infrequently employed as enantioinduction components in stereoselective catalysis. In this review, synthetic approaches to carbohydrate-based ligands and catalysts are outlined, along with example applications in enantioselective catalysis. A wide range of carbohydrate-based functionality is covered, and key trends and future opportunities are identified.

## Abstract 2

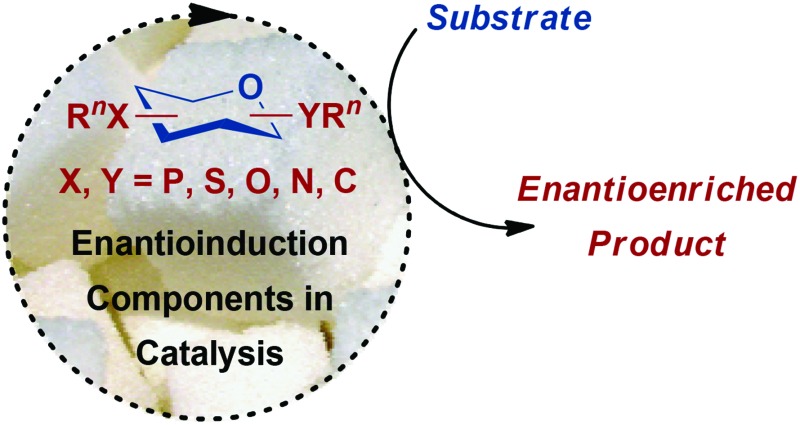
In this review, synthetic approaches to carbohydrate-based ligands and catalysts are outlined, along with example applications in enantioselective catalysis.

## 1. Introduction

Enantioselective catalysis has become the dominant approach to the asymmetric synthesis of chiral molecules. Relaying “chiral information” from a sub-stoichiometric source, by way of a useful chemical transformation, will underpin future advances in asymmetric chemistry.^1^ The development of new chiral metal–ligand complexes and organocatalysts, which surpass established enantioinduction benchmarks or provide new enantioselective transformations, is of huge importance because of the vital role of homochiral molecules in drug design. A large proportion of chiral catalysts are prepared directly from biologically derived sources (*e.g.* amino acids). Carbohydrates are a class of abundant and readily modifiable “chiral pool” building blocks. However, these stereochemically rich biomolecules continue to be underexploited in catalyst design.^2^ This represents something of a chemical paradox, which has not arisen through lack of effort from synthetic chemists. Many reports describe the use of carbohydrates in stereoselective transformations, and this area has been reviewed previously.^3^ A preconception may exist that carbohydrates are challenging to work with, and therefore largely limited to glycobiological applications.^4^ However, carbohydrates are innately chiral and possess a valuable array of stereochemistry, functionality and scaffold diversity, which can all be exploited in catalyst design.

**Fig. 1:**
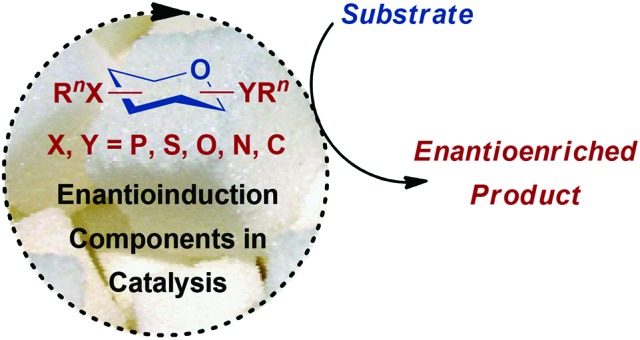
Carbohydrates as enantioinduction components.

This review aims to break the stigma associated with carbohydrate chemistry, by highlighting expedient, modular and diversifiable synthetic routes to carbohydrate-based catalysts for application in asymmetric organo- and transition-metal (TM) catalysis (Fig. 1). It is our hope that the successful strategies outlined here will stimulate the design and evaluation of novel carbohydrate-based systems in other areas of asymmetric catalysis. Although by no means exhaustive, the examples discussed herein contain key steps towards the ligand or catalyst, along with benchmark applications in enantioselective catalysis.^5^

## 2. Selected examples

### 2.1 Phosphine ligands

**Scheme 1:**
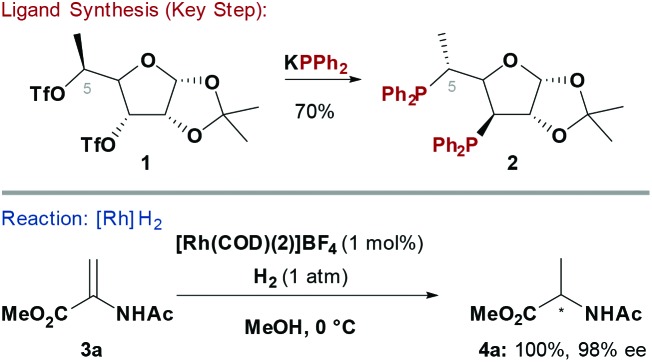
Synthesis and evaluation of a PR_3_ ligand.

**Scheme 2:**
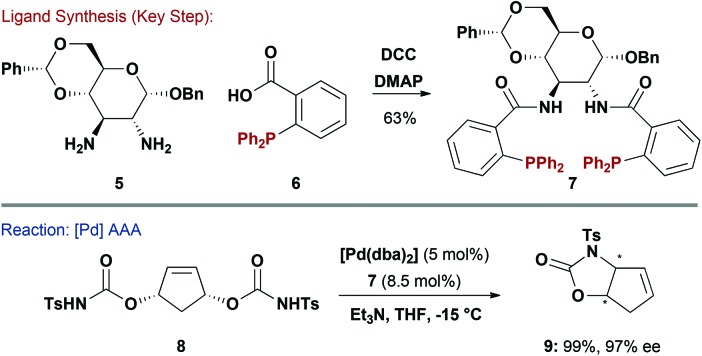
Synthesis and evaluation of a PR_3_ ligand.

Chiral phosphine ligands have underpinned progress in asymmetric TM catalysed reactions.^6^ Early homochiral bidentate phosphines, such as 2,3-*O*-isopropylidene-2,3-dihydroxy-1,4-bis(diphenylphosphino)butane (DIOP),^7^ used chiral pool building blocks to construct the ligand backbone and this strategy has been extensively explored. Two main approaches have been developed to access carbohydrate-based systems embodying a P–C_carbohydrate_ bond: (1) S_N_2 displacement of activated alcohols by P-based nucleophiles^8^ (Scheme 1) and (2) nucleophilic opening of anhydro-sugars^9^ (see Scheme 5). A complication often observed with the former approach is competing E2 elimination.

Diéguez and co-workers showed that furanoside-derived ligand **2** could be synthesised by double S_N_2 displacement of ditriflate **1** with KPPh_2_ (Scheme 1).^10^ Chiral phosphine **2** was effective for Rh-catalysed enantioselective hydrogenations of “classical” chelating alkene substrates. For example, reduction of methyl 2-acetamidoacrylate (**3a**) proceeded with excellent levels of enantioselectivity (98% ee) at 0 °C to deliver **4a** in quantitative yield. Notably, the C-5 methyl group of **2** was critical to the efficiency of the process. In the absence of this substituent low conversions were observed, whereas the corresponding C-5 epimer of **2** provided significantly lower levels of enantioselectivity.^10,11^ The synthetic route to **2** is modular, such that further variations of ligand structure can be easily envisaged by varying the P-based nucleophile or the furanoside substituents.

Ligand systems where the phosphine is not directly attached to the carbohydrate unit have also been reported. For example, Ruffo and co-workers accessed **7** by amide coupling of carboxylic acid containing phosphine **6** with protected 2,3-glucodiamine **5** (Scheme 2).^12^ Given that **7** closely resembles the highly successful Trost Ligand,^13^ it was unsurprising that it performed efficiently in Pd-catalysed asymmetric allylic alkylation (AAA) reactions. For example, desymmetrisation of **8** proceeded smoothly to generate carbamate **9** in excellent yield and 97% ee. Carbohydrate diamine **5** could be considered “greener” than *trans*-diaminocyclohexanes commonly used for Trost ligand synthesis because the latter require resolution to access enantiopure material. A d-mannose derived pseudo-enantiomeric variant of **7** was also reported.^12^ It is pertinent to note that diamine **5** has also been used in the synthesis of salen ligands for Mn-catalysed epoxidation of styrenes.^14^

### 2.2 Phosphinite ligands

**Scheme 3:**
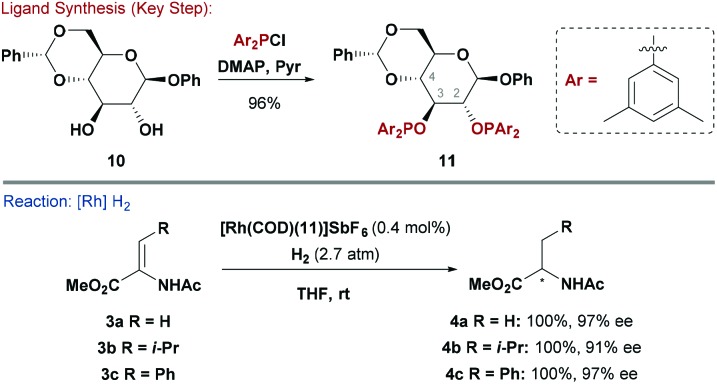
Synthesis and evaluation of a R_2_POR ligand.

P–O bonds are generally easier to form than P–C variants and this enables the direct attachment of carbohydrate scaffolds to phosphorous centres (*cf.* Scheme 1).^3*b*^ In early work, Selke synthesised a range of hexapyranoside-derived phosphinites and examined how their configuration affected enantioselectivity in Rh-catalysed asymmetric hydrogenations of functionalised alkenes. It was found that systems with all equatorial substituents (*e.g.* β-glucose derivatives) were most effective.^15^ Later, RajanBabu and co-workers exploited the potential of modular P–O bond formation to synthesise a library of phosphinite ligands by reacting β-glucoside-based backbone **10** with electronically distinct chlorodiarylphosphines (Scheme 3).^16^ For the reduction of dehydroamino ester precursors **3**, electron rich P(aryl)_2_ groups induced the highest levels of asymmetry. For example, modification of a cationic Rh pre-catalyst with ligand **11** provided a system that was effective for a wide range of β-aryl substituted amino esters (*e.g.* **4c**, 97% ee). For alkyl substituted systems, enantioselectivity exhibited a greater substrate dependency, but, nevertheless, **4a** (97% ee) and **4b** (91% ee) were both accessed in an efficient manner. It is pertinent to note that a 3,4-*O*-diphosphinite glucopyranoside functioned as a pseudo-enantiomeric version of **11**.^16^ Similar ligand systems have been evaluated in asymmetric Ni-catalysed hydrocyanations of vinyl-arenes.^17^

### 2.3 Phosphite ligands

**Scheme 4:**
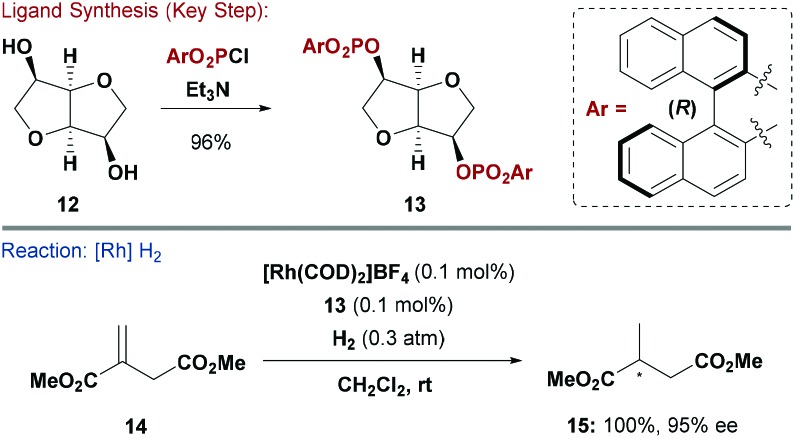
Synthesis and evaluation of a P(OR)_3_ ligand.

The facile synthesis of P–O bonds has also enabled the modular construction of phosphite ligands from carbohydrate alcohols and diols. An early example by Reetz and co-workers relied on reaction of isomannide (**12**), which has a rigid concave structure, with various diaryl phosphorochloridates to furnish bidentate ligands such as **13** (Scheme 4).^18^ Phosphite **13** was evaluated in Rh-catalysed asymmetric hydrogenation of dimethyl itaconate (**14**) and afforded **15** in high ee (96%). The alternate (*S*)-BINOL derivative of **13** functioned as an efficient pseudo-enantiomeric ligand (**15**: 87% ee). Interestingly, ligands derived from achiral diaryl phosphorochloridates and **12** also induced high levels of asymmetry (see below). The option of modifying **12** with stereodefined diaryl phosphorochloridates is a useful strategy to fine-tune asymmetric induction and control product stereochemistry. Reetz and co-workers also showed that mono-phosphite ligands based on a similar scaffold to **13** are useful ligands for enantioselective metal catalysed transformations.^19^

In a complementary approach by the groups of Diéguez and Claver, glucose derived furanoside diols, featuring a range of stereochemistries, were reacted with axially chiral diaryl phosphorochloridates to give a range of carbohydrate-based diphosphites in a modular manner.^20^ These ligands were evaluated in Rh-catalysed hydroformylation of styrenes giving branched aldehydes in up to 98% regioselectivity and 91% ee.

### 2.4 Mixed P ligands

**Scheme 5:**
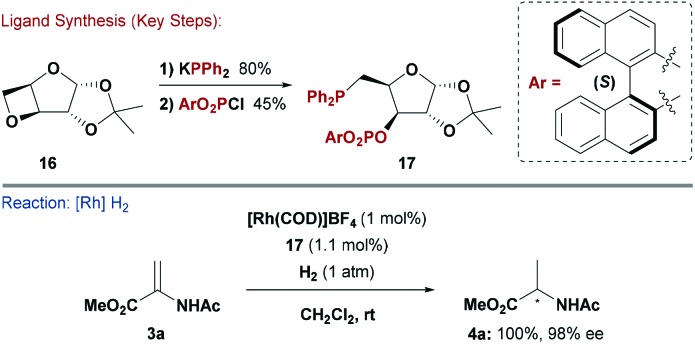
Synthesis and evaluation of a P(OR)_3_-PR_3_ ligand.

Given the wide range of methods discussed above, it is not surprising that several mixed P-bidentate carbohydrate ligand systems have been developed. The sheer number of possible variations is high, hence only one example is illustrated below. Ruiz and co-workers reported phosphine–phosphite ligands in an attempt to merge favourable characteristics (*e.g.* high ee and catalyst turnover frequency (TOF)) found in the individual catalytic systems (Scheme 5).^21^ There has been growing demand for chiral electron deficient P-based ligand systems,^22^ and carbohydrate building blocks can provide facile access to a wide range of variants.

The Ruiz approach hinged on derivatising modified d-furanoside xylose core **16**. The PR_3_ unit was installed by oxetane ring opening of **16** with KPPh_2_. This step released a hydroxyl group, which could then be exploited for P–O bond formation by reaction with a range of diaryl phosphorochloridates.^21^ A Rh-system modified with **17** gave high enantioselectivities in the hydrogenation of various substituted acetamidoacrylates. Similarly to studies by Reetz and co-workers,^18^ it was found that the stereochemistry of product (**4a**) was controlled by the axial chirality of the (*S*)- or (*R*)-BINOL motif in **17**. However, again, systems derived from achiral biaryls (in place of BINOL) were also efficient, presumably because the chirality of the carbohydrate backbone controlled the conformation of this unit.^21^ Studies by the groups of Claver and Diéguez utilised similar synthetic steps to access bidentate phosphite–phosphoramidite and diphosphoramidate ligands. These showed good results in asymmetric Rh-catalysed hydrogenations of chelating alkene substrates.^23^

### 2.5 P,alkene ligands

Due to their affinity for metal centres through cooperative binding modes, alkenes are widely employed as substrates in late-TM catalysis. This property has also stimulated the development of chiral diene and P,alkene ligands.^24^ Unsaturated carbohydrates are readily available (*e.g.* glycals and 2,3-unsaturated glycosides) and Boysen and co-workers have endeavoured to exploit these feedstocks for the easy synthesis of P,alkene ligands. These systems are of use for a variety of asymmetric reactions, including Rh-catalysed 1,4-additions of arylboronic acids to enones.^25–28^

**Scheme 6:**
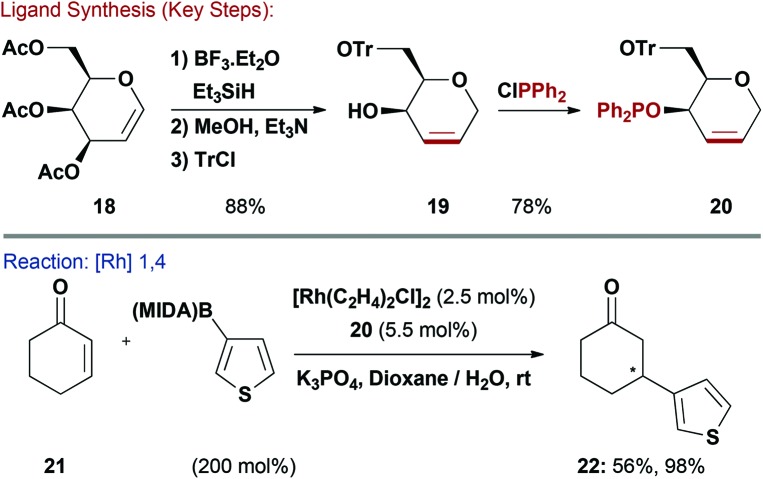
Synthesis and evaluation of a R_2_POR-Alkene ligand.

Ferrier rearrangement of commercially available acetylated galactal **18**, followed by global deprotection and selective tritylation (Tr) of the primary alcohol gave **19** in good overall yield (Scheme 6). Reaction of the allylic alcohol of **19** with ClPPh_2_ effected P–O bond formation to generate phosphinite-alkene ligand **20**.^26^ This was evaluated in challenging Rh-catalysed asymmetric additions of heteroaryl MIDA and pinacol boronates to cyclohexenone (**21**).^28^ Excellent enantioselectivities were observed using a wide range of heteroaryl nucleophiles, albeit often in modest yield. For example, thiophene derivative **22** was delivered in 56% yield and 96% ee. The modularity of the ligand synthesis and the availability of different carbohydrate precursors allowed Boysen and co-workers to conduct extensive structure-activity studies, which also led to pseudo-enantiomeric ligand systems.^26,27^

### 2.6 P,N ligands

Chiral mimics of Crabtree's catalyst have been highly successful for enantioselective hydrogenation of unfunctionalised olefins, and numerous other asymmetric transformations now use P,N ligands.^29^ The possible permutations of P and N donors in these kind of systems is large. For example the P-based unit can be a phosphine, phosphinite or phosphite, whereas the N-donor is often part of an oxazoline or a pyridine.^30^ The *trans*-effect that such systems impart when ligated to a metal is crucial to their efficacy in enantioselective processes.^31^ Ohe, Uemura and co-workers reported carbohydrate P,N ligands based on a glucosamine derived scaffold. Their system offers high structural flexibility as both the oxazoline heterocycle and P-based substituents can be easily modified.^32^

**Scheme 7:**
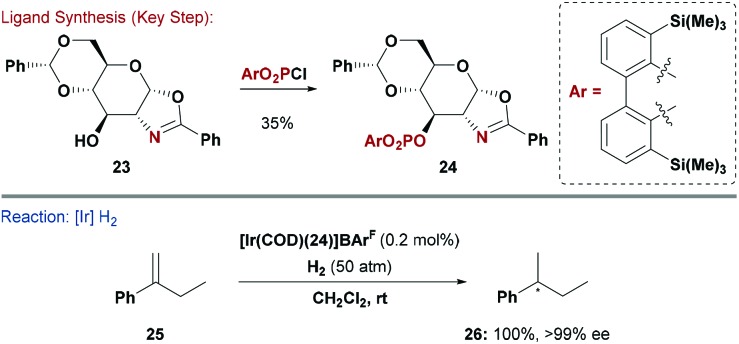
Synthesis and evaluation of a P,N ligand.

The groups of Diéguez and Andersson capitalised on this approach and synthesised a series of phosphite-oxazoline ligands, which were evaluated in Ir-catalysed hydrogenations of unfunctionalised olefins (Scheme 7).^33^ A range of substituted *gluco*-oxazolines (*e.g.* **23**) were reacted with diaryl phosphorochloridates to give the corresponding P,N ligands (*e.g.* **24**). By combining these with a cationic Ir-source, excellent enantioselectivities could be obtained for the hydrogenation of a broad range of tri- and 1,1-di-substituted olefins. Critical to the success of this approach was the ability to rapidly modify both the P and N donors. Ligand **24** and related derivatives are also effective in asymmetric Heck reactions.^34^

### 2.7 S-based ligands

**Scheme 8:**
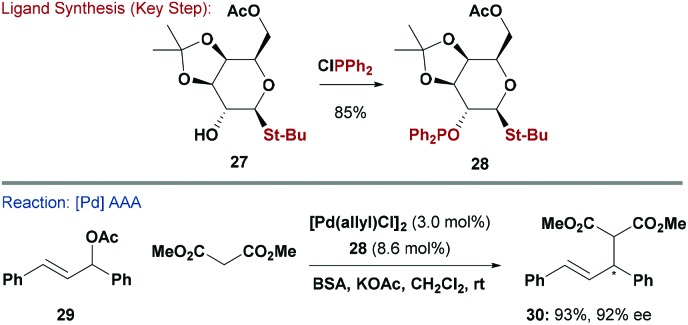
Synthesis and evaluation of a P,S ligand.

In a similar vein to P,N ligands, P,S ligands have been evaluated in a wide range of asymmetric processes.^3*b*^ The incorporation of S functional groups into carbohydrates is well established due to the synthetic importance of thioglycosides as glycosyl donors for oligosaccharide synthesis.^35^ Glycosylation with thiols is very general and provides a modular approach to diversifying any potential ligand library. In this context, Khiar and co-workers synthesised a range of mono-hydroxylated thio-galactopyranosides (*e.g.* **27**) and then used the free-OH to give a series of chiral thio-phosphinite ligands (*e.g.* **28**) (Scheme 8).^36^ Interestingly, ligation of **28** to a Pd-centre afforded selectively a single diastereomer, resulting from preferential coordination of one of the two diastereotopic sulfur lone pairs.^36,37^ This system was evaluated in Pd-catalysed AAA of dimethylmalonate with (*E*)-1,3-diphenylallyl acetate (**29**) and gave **30** in high yield and 92% ee. The synthesis of a pseudo-enantiomeric version of **28**, where a d-arabinose core was used to invert the stereo-relationship between the S and P groups, was also described.^36^ Both systems were also evaluated in asymmetric Rh-catalysed hydrogenations of enamides.

In a related study, Khiar and co-workers glycosylated dithiols with peracylated glycosyl donors to give chelating *C*_2_-symmetric carbohydrate-bis(thioether) ligands, which were evaluated in Pd AAA.^38^ Additionally, Pregosin and co-workers have reported carbohydrate derived S-oxazoline ligands. These were accessed by alkylating anomeric thiols with oxazoline-based alkyl chlorides. These systems afforded high enantioselectivities in Pd-catalysed AAA reactions.^39^

**Scheme 9:**
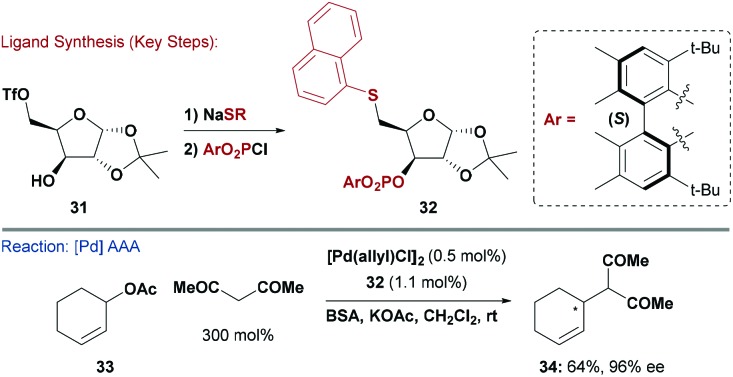
Synthesis and evaluation of a P,S ligand.

An alternative strategy by Pàmies, Diéguez and co-workers exploited the facile displacement of the primary alkyl triflate of d-xylose derivative **31** with thiolate nucleophiles to create a library of furanoside-based thioether ligands (Scheme 9).^40^ The secondary alcohol of the product was then utilised for P–O bond formation to give various thio-phosphite ligands (*e.g.* **32**). This highly modular approach led to the discovery that ligand **32** is useful for asymmetric Pd-catalysed C–C, C–N and C–O bond formations between allylic acetates and various nucleophiles. Notably, **32** could induce high asymmetry for processes involving both cyclic (*e.g.* **33**) and acyclic allylic acetates.

### 2.8 Oxazoline ligands

*C*_2_-Symmetric bis(oxazoline) ligands have revolutionalised asymmetric Lewis acid and TM catalysed reactions. This ligand class is especially popular due to its modularity and synthetic accessibility.^41^ Most *C*_2_-symmetric chiral oxazoline ligands use amino acid derived 1,2-amino-alcohols as the source of chirality. Surprisingly, amino-sugars, such as glucosamine, have not been widely exploited in the synthesis of this ligand class.^42^

**Scheme 10:**
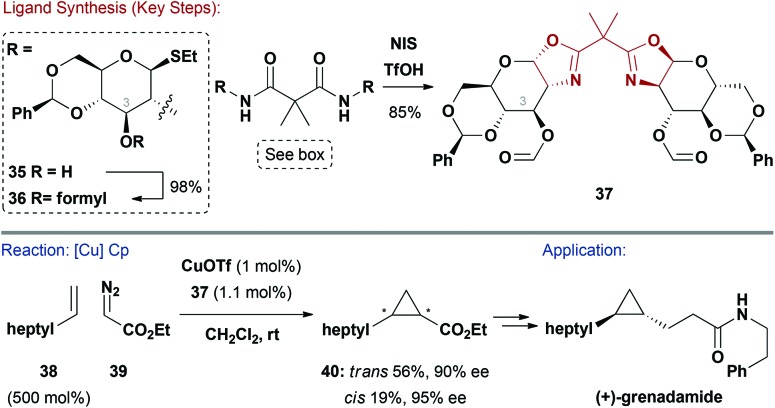
Synthesis and evaluation of a bis(oxazoline) ligand.

Boysen and co-workers have published extensive studies on a series of bis(oxazoline) ligands such as **37** (Scheme 10).^42,43^ The nature of the C3–OH appendage impacted asymmetric induction during catalysis and inversion or removal of this group gave decreased selectivities.^43*b*^ One synthetic route facilitated late-stage modification of derivative **35** at the crucial C3–OH with a variety of groups (Ac, Piv, Me, Bn *etc.*), such as formyl (Scheme 10). Treatment of **36** with NIS and TfOH resulted in bis(oxazoline) **37**, *via* double cyclisation of the amides.^43*b*^ A complex formed *in situ* between **37** and CuOTf was efficient in catalysing the asymmetric cyclopropanation (Cp) of styrenes with diazoacetates.^42,43^ One of the more challenging processes, involving aliphatic alkene **38** and diazoacetate **39**, proceeded smoothly to deliver **40** in 75% yield and 90% ee for the *trans*-diastereomer (73 : 27 *trans* : *cis*). This intermediate could be further elaborated to (+)-grenadamide.^44^ Related carbohydrate pybox and thiazoline ligands have also been explored, with the former delivering high enantioselectivities in Cu-catalysed alkynylations of imines.^45^

### 2.9 N-heterocyclic carbene (NHC) ligands

NHCs have rapidly found wide-ranging applications in organo- and TM-catalysed transformations.^46^ In particular, chiral variants of this ligand class have been extensively researched for asymmetric processes.^47^ These studies have included systems where carbohydrates serve as the source of homochirality.

**Scheme 11:**
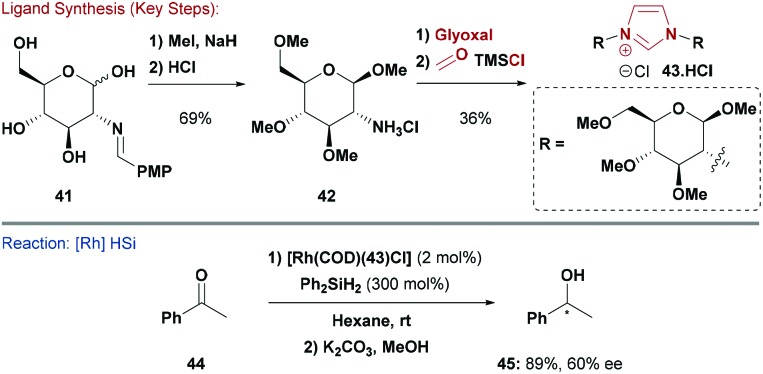
Synthesis and evaluation of an NHC ligand.

Early approaches to carbohydrate-based NHC ligands exploited glycosylation of imidazoles with pyranoside-based anomeric bromides to give un-symmetrical C-1 linked NHCs. These were evaluated in alkene metathesis reactions and organocatalysis.^48^ However, this approach was limited as there were few options for modification of the carbohydrate hydroxyls. An alternative approach developed by Henderson, Bower and Galan, used glucosamine derivative **41**, which could be *O*-alkylated with a variety of different groups, thereby providing a short and diversifiable approach to carbohydrates such as **42** (Scheme 11).^49^ Conversion of **42** to *C*_2_-symmetric imidazolium **43** was readily achieved, and ligation to Rh proceeded smoothly. The resulting complex showed promising results in asymmetric hydrosilylations of ketones (*e.g.* **44**). For example, alcohol **45** was obtained in 89% yield and 60% ee.^49^ The effects of carbohydrate stereochemistry were also examined using related mannosamine derived NHC ligands, but these provided inferior results.

**Scheme 12:**
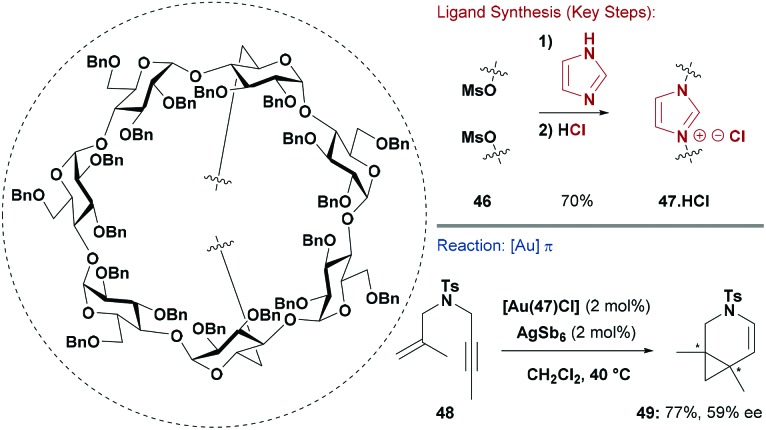
Synthesis and evaluation of a cyclodextrin-based NHC ligand.

An alternate and very elegant strategy was developed by Sollogoub and co-workers who selectively synthesised β-cyclodextrin **47·HCl** by S_N_2 displacement of parent bis-mesylate **46** with imidazole (Scheme 12).^50^ A similar bis-alkylation with benzimidazole provided access to a core modified variant. The Ag-complex of **47** was used to transmetallate the NHC onto AuCl, and the resulting system was applied to an asymmetric Au-catalysed enyne cyclisation/cyclopropanation reaction, which gave **49** in 77% yield and 59% ee.^50^

### 2.10 O-based ligands

Alkoxides generate hard anions and so their deployment as ligands for late-TMs is somewhat limited. However, when used in conjunction with oxophilic metals, the resulting Lewis acidic (LA) or basic (LB) complexes can be versatile catalysts for chiral transformations.^51^

**Scheme 13:**
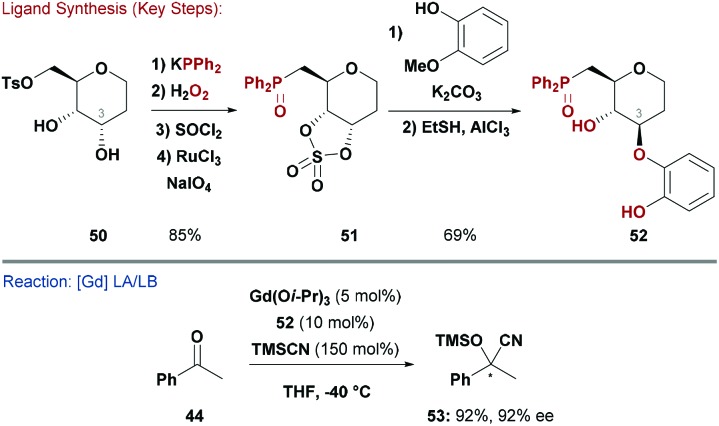
Synthesis and evaluation of an O ligand.

Shibasaki and co-workers synthesised a range of deoxy-glucose-based ligands (*e.g.* **52**) which possess free hydroxyl groups.^52^ Initially, the catechol moiety in **52** was installed by S_N_Ar substitution of a Cr-complexed fluoro-arene.^52a,b^ However, this approach did not allow modular access to a ligand library so an alternate sequence was employed. Reduction of d-glucal, inversion of the C3–OH and formation of the C6–OTs gave deoxy-allose scaffold **50** ready for functionalisation (Scheme 13). This was converted to cyclic sulfate **51**, which could be opened stereospecifically with different catechol derivatives.^52*c*^ The ligand class was designed for TM- or lanthanide-catalysed asymmetric cyanosilylation of ketones (Scheme 13).^52^ Indeed, catalytic quantities of **52** and Ti(Oi-Pr)_4_ effected 1,2-addition of TMSCN to acetophenone to deliver product **(*R*)-** **53** in 85% yield and 92% ee. An analogous reaction, using a 2 : 1 ratio of **52** and Gd(Oi-Pr)_3_ as the catalyst, gave **(*S*)-** **53** in 92% yield and 92% ee.^53^ The combination of a bimetallic Gd complex with **52** was far more reactive than the corresponding Ti system, and has since been employed in enantioselective Strecker reactions of ketoimines,^54^ 1,4-additions of cyanide to α,β-unsaturated *N*-acylpyrroles,^55^ and desymmetrisations of *N*-acyl aziridines with TMSCN.^56^

### 2.11 N-based ligands

Homochiral 1,2-amino-alcohols can be used to construct other ligand classes, such as oxazolines, or one can directly exploit the ligation ability of the NH and OH groups in TM catalysis. This approach has seen widest application in asymmetric transfer hydrogenation (ATH).^57^

**Scheme 14:**
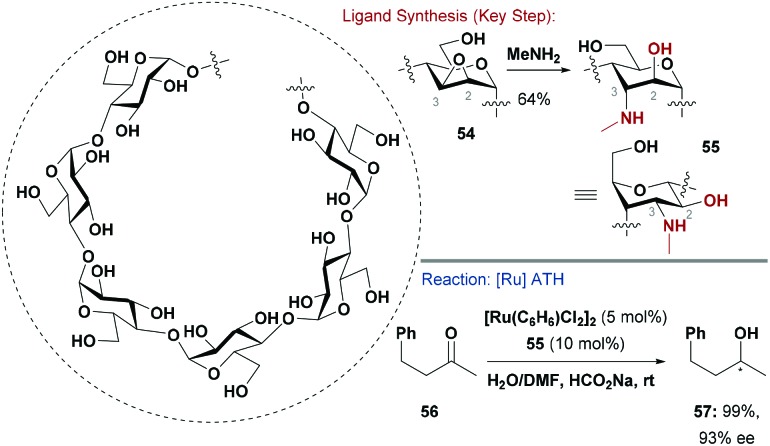
Synthesis and evaluation of an N,O cyclodextrin-based ligand.

Woggon and co-workers reported several β-cyclodextrin derived 1,2-amino-alcohol ligands for Ru-catalysed ATH of challenging alkyl–alkyl ketones (Scheme 14).^58^ Ligand **55** was accessed by regioselective opening of epoxide **54** with methylamine (other amines could also be used).^58*b*^ ^1^H NMR indicated that the resulting *anti*-relationship between the C-2 and C-3 substituents caused carbohydrate **55** to ring flip to the indicated conformer. The C-3*N*H and C-2*O*H of **55** ligated to [Ru(C_6_H_6_)Cl_2_]_2_, and the resulting homochiral complex effected high asymmetric induction in ketone reduction; for example, reduction of **56** generated **57** in 93% ee. The efficacy of the system was attributed to encapsulation of the ketone substrate within the cyclodextrin pore.^58*b*^

**Scheme 15:**
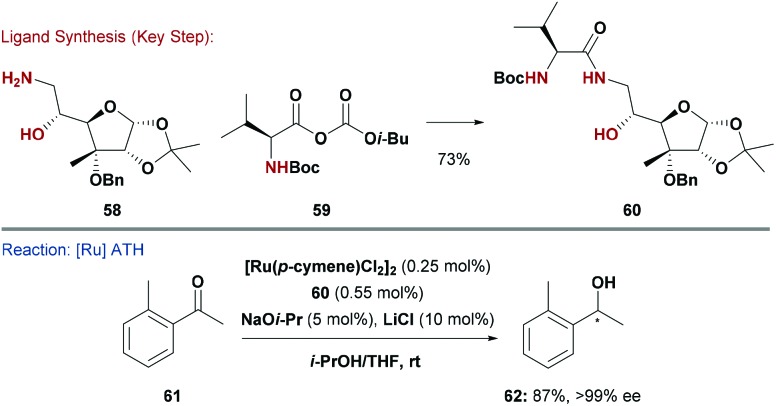
Synthesis and evaluation of an N,N,O ligand.

In an alternate strategy, the groups of Diéguez and Adolfsson pursued a modular approach to a new family of N,heteroatom donors. Here, carbohydrate derived amines were *N*-acylated with protected amino acids to give hydroxy-amide type ligands, such as **60** (Scheme 15).^59^ The C-6 amine of **58** was installed by reduction of the corresponding azide, itself accessed by S_N_2 displacement of a primary tosylate. The modularity of the approach allowed the introduction of different amino-acid derivatives (*e.g.* **59**) with varying steric demands and stereochemistry. Ligand **60** was used in Ru-catalysed ATH of aryl–alkyl ketones (*e.g.* **61**), and delivered the product alcohols in very high enantioselectivity (generally >99% ee), including sterically hindered variants, such as **62**. Interestingly, the carbohydrate unit of **60** is primarily responsible for asymmetric induction. A system in which the valine unit was replaced with a glycine residue still gave high enantioselectivities, albeit with lower catalytic activity.^59^

**Scheme 16:**
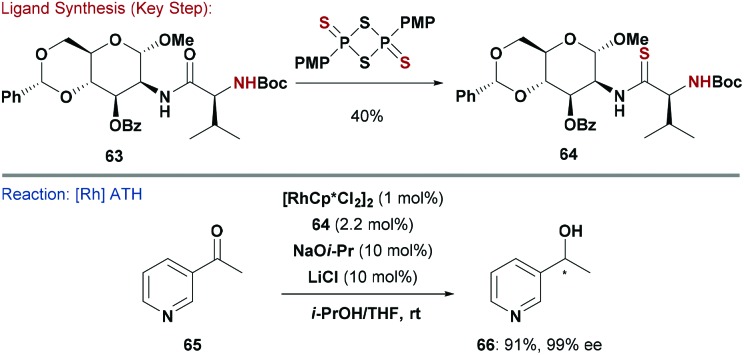
Synthesis and evaluation of an N,S ligand.

Another popular ligand class for asymmetric ATH are thioamides (*e.g.* **64**, Scheme 16), which can be accessed by treatment of parent amide **63** with Lawesson's reagent. Diéguez and co-workers explored furanoside and pyranoside thioamides, using their previous strategy^59^ of coupling amino acids with carbohydrates (Scheme 15).^60^ This modular approach led to the identification of mannosamine-(*S*)-valine **64** as an efficient ligand for Rh-catalysed ATH of heteroaromatic ketones (*e.g.* **65**).^60*c*^ Using this system, alcohol **66** was generated in high yield (90%) and enantioselectivity (99%). Interestingly, the (*R*)-valine derived diastereomer of **64** functioned efficiently as a pseudo-enantiomeric ligand.

**Scheme 17:**
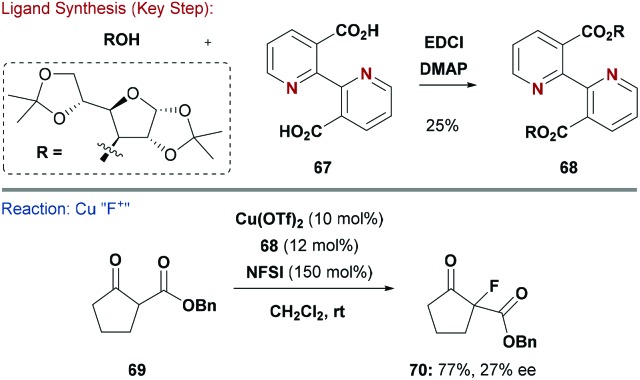
Synthesis and evaluation of a bipyridine ligand.

Carbohydrate-modified pyridine ligands are sparsely documented, even though scaffolds such as bypridine (bpy) are ubiquitous in many TM catalysed protocols.^61^ An interesting example from Billard, Queneau and co-workers describes the synthesis of bipyridine-diesters (*e.g.* **68**) from the parent carboxylic acid, **67**, and various carbohydrate alcohols, such as diacetone glucose (Scheme 17).^62^ When **68** was combined with Cu(OTf)_2_, β-keto ester **69** underwent electrophilic fluorination with NFSI to give products, such as **70**, in moderate yields and low enantioselectivity (27%). These results suggest that this approach might have further potential for optimisation.

### 2.12 Organocatalysts

Asymmetric organocatalysis has emerged as a powerful strategy to access enantioenriched molecules.^46c,63^ The well-established Shi epoxidation system has been the focus of several in-depth reviews and will not be discussed further.^5b,c^ H-bond donors, such as (thio)ureas, have proved immensely popular since their inception.^64^ A wide variety of chiral pool building blocks have been evaluated as the source of chirality for these systems. A seminal report by Kunz and co-workers described the use of carbohydrate-based urea organocatalysts in asymmetric Strecker and Mannich reactions.^65^ Later, carbohydrate based primary-amine thiourea organocatalysts were also developed. For example, Ma and co-workers disclosed systems for enantioselective addition of aromatic ketones to nitro-olefins.^66^ Independently, Zhou and co-workers developed similar tertiary amine-thiourea catalysts for asymmetric aza-Henry reactions.^67^ The discovery of these carbohydrate-based amine thiourea organocatalysts was facilitated by their modular construction.^66,67^

**Scheme 18:**
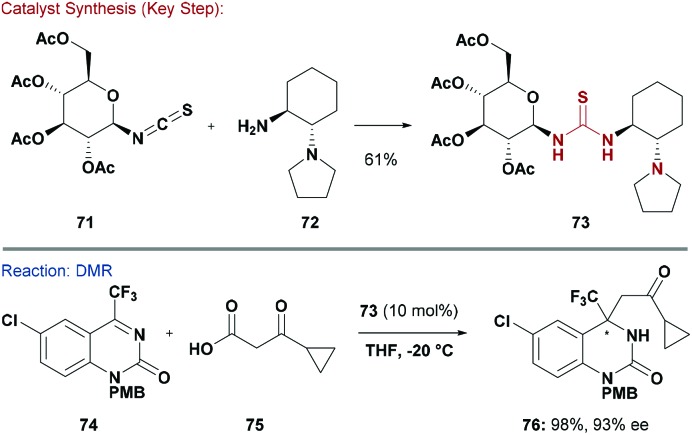
Synthesis and evaluation of a thiourea organocatalyst.

To highlight an example, the addition of functionalised chiral amine **72**, serving as the variational motif, to anomeric thioisocyanate–carbohydrate **71** delivered thiourea **73** (Scheme 18).^66^ Ma and co-workers applied organocatalyst **73** to a decarboxylative Mannich reaction (DMR) between β-keto acids (*e.g.* **75**) and ketimines (*e.g.* **74**). This afforded products such as **76** in high yields (98%) and enantioselectivities (93%).^68^ Elaboration of **76** into anti-HIV drug DPC 083 was also reported. However, organocatalyst **73**, derived from common d-glucose, gave the incorrect enantiomer and so the antipode of thiourea **73** was used. In a related Mannich reaction, the structural effects of the carbohydrate unit were probed and found to be crucial. In the absence of this unit, the levels of asymmetric induction were inferior.^69^

It is pertinent to note that several carbohydrate-based amines have been developed for enamine catalysis. Benchmark applications have focussed on aldol reactions between cyclohexanone or acetone and electron deficient aryl aldehydes.^70^

**Scheme 19:**
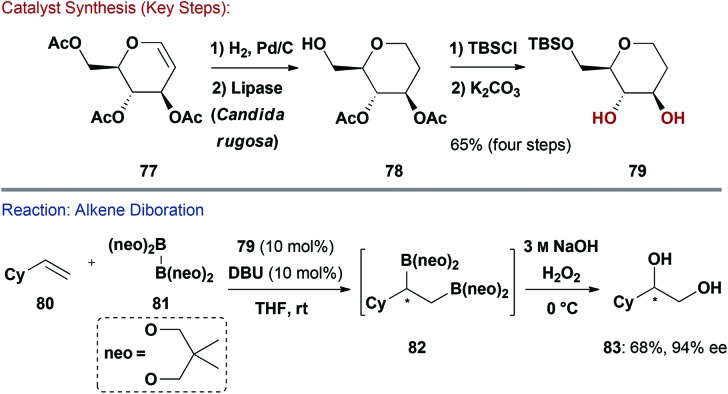
Synthesis and evaluation of a diol organocatalyst.

More recently Morken and co-workers have reported enantioselective diborations of alkenes catalysed by de-oxy carbohydrates (Scheme 19).^71^ Key diol **79** was synthesised in 4-steps in 65% yield on a gram scale from commercially available d-glucal **77**. Hydrogenation of the double bond followed by enzyme-mediated O-6 selective deacetylation afforded **78**. Silylation of the free hydroxyl group and then global deacetylation gave **79** concisely. Given the wide range of silyl protecting groups available, it is easy to envisage modular access to derivatives of the diol catalyst. By exploiting the increased reactivity of the homochiral diboron reagent formed from the reversible displacement of the neopentyl (neo) ligands in **81** with **79** ((**79**)_2_B_2_), the enantioselective diboration of alkenes (*e.g.* **80**) was achieved in high ee's and good yields. The intermediate boronic esters (*e.g.* **82**) can undergo stereospecific oxidation to give the corresponding diol (*e.g.* **83**), or, alternatively, can be modified by site selective Pd-catalysed cross-coupling reactions (not shown). It is pertinent to note that an l-rhamnal-derived diol functioned as an efficient pseudo-enantiomeric catalyst.^72^

## 3. Conclusions and outlook

The aim of this review is to exemplify synthetic routes to carbohydrate derivatives that have been used as enantioinducing components in catalysis. This survey reveals the frequent use of glucosyl scaffolds, in both the furan- and pyranoside forms, alongside a plethora of C–X (C–S, C–N and C–P), O–P and N–P bond formations. The resulting “toolbox” of methods provides flexible entries to a wide range of normally bidentate ligand systems. The carbohydrate unit can also be combined with other readily available homochiral sources, such as amino acids or conformationally restricted biaryls. This approach provides a high degree of modularity and often easy access to pseudo-enantiomeric systems.

Because carbohydrate chemistry has evolved as a methodology for oligosaccharide synthesis, it is not surprising that common protecting groups (esters, acetals and alkyl ethers) are routinely featured. However, for chiral catalyst systems, the C–OH functionality provides an opportunity for modification or tuning, rather than simply requiring protection. For example, “clicking” in aryl groups by S_N_Ar would deliver carbohydrate–aryl ethers, where sterics and electronics could be varied to produce ligand/catalyst libraries in a rapid manner.^2*c*^

Although access to the carbohydrate-based systems in the examples described here is generally expedient, the asymmetric reactions chosen for evaluation are largely benchmarks and not new transformations. Applications to asymmetric hydrogenation of chelating olefins or AAA reactions addresses a mature field. The deployment of carbohydrate-based catalysts in asymmetric methodologies that embody novel or underutilised reactivity modes would greatly elevate their importance in asymmetric catalysis.
